# New insights into acupuncture techniques for poststroke spasticity

**DOI:** 10.3389/fpubh.2023.1155372

**Published:** 2023-04-06

**Authors:** Jun-Xiang Wang, Olivia Lai Fidimanantsoa, Liang-Xiao Ma

**Affiliations:** ^1^School of Nursing, Beijing University of Chinese Medicine, Beijing, China; ^2^International School, Beijing University of Chinese Medicine, Beijing, China; ^3^School of Acupuncture-Moxibustion and Tuina, Beijing University of Chinese Medicine, Beijing, China; ^4^The Key Unit of State Administration of Traditional Chinese Medicine, Evaluation of Characteristic Acupuncture Therapy, Beijing, China

**Keywords:** stroke, spasticity, motor dysfunction, acupuncture technique, rehabilitation, non-pharmacological intervention, review, clinical and experimental evidences

## Abstract

With the trend of aging population getting more obvious, stroke has already been a major public health problem worldwide. As a main disabling motor impairment after stroke, spasticity has unexpected negative impacts on the quality of life and social participation in patients. Moreover, it brings heavy economic burden to the family and society. Previous researches indicated that abnormality of neural modulation and muscle property corelates with the pathogenesis of poststroke spasticity (PSS). So far, there still lacks golden standardized treatment regimen for PSS; furthermore, certain potential adverse-events of the mainstream therapy, for example, drug-induced generalized muscle weakness or high risk related surgery somehow decrease patient preference and compliance, which brings challenges to disease treatment and follow-up care. As an essential non-pharmacological therapy, acupuncture has long been used for PSS in China and shows favorable effects on improvements of spastic hypertonia and motor function. Notably, previous studies focused mainly on the research of antispastic acupoints. In comparison, few studies lay special stress on the other significant factor impacting on acupuncture efficacy, that is acupuncture technique. Based on current evidences from the clinic and laboratory, we will discuss certain new insights into acupuncture technique, in particular the antispastic needling technique, for PSS management in light of its potential effects on central modulations as well as peripheral adjustments, and attempt to provide some suggestions for future studies with respect to the intervention timing and course, application of acupuncture techniques, acupoint selection, predictive and aggravating factors of PSS, aiming at optimization of antispastic acupuncture regimen and improvement of quality of life in stroke patients. More innovations including rigorous study design, valid objective assessments for spasticity, and related experimental studies are worthy to be expected in the years ahead.

## Introduction

Stroke is a critical public health problem worldwide and is the leading cause of death in China (1). As the most common complication poststroke, spasticity is reconsidered as velocity- and muscle length- dependent increase in resistance to externally imposed muscle stretch, which can be explained by hyperexcitable descending excitatory brainstem pathways and secondary exaggerated stretch reflex responses (2). The prevalence of poststroke spasticity (PSS) ranges from 17% to 43% during the first year, and approximately two-thirds of patients with spasticity have both upper and lower extremities involvement (3, 4). After lesions to the cerebral cortex and its descending pathway (cortico-spinal tract, CST), weakness occurs immediately, while spasticity emerges and develops later (2). Spasticity interacts with weakness, resulting in disabling motor impairments and complex complications like muscle contracture, motor dysfunction and spastic pain, which negatively impacts on patients’ quality of life (5, 6). The direct costs of PSS patients are four times to those without spasticity, imposing heavy financial burdens to the family and society (7). Nowadays, a wide range of treatment options are available, including oral drugs, physical techniques, botulinum toxin injection and surgery to target central and/or peripheral factors (8). However, certain unpredictable adverse-events such as drug-induced hepatorenal toxicity and generalized muscle weakness, and high risks related surgery still remain problematic (9–11). Therefore, a safer, effective and a more economical treatment modality is desired by both doctors and patients.

Acupuncture has long been used for treating PSS in China. The incidence of adverse-effects in this ancient art of healing technique is much lower than that of western medicine (12). As we know, there are two major factors determining the efficacy of acupuncture, one is acupoint selection, the other acupuncture technique. Apparently, majority of previous studies focused mainly on acupoint research, committed to finding out the most effective antispastic acupoint or acupoint combinations. For example, in views of traditional Chinese medicine (TCM) theory, acupoints either target to regulate disordered brain functions, such as scalp acupoints/lines (e.g., MS6-parietal anterior temporal oblique line, and MS7-parietal posterior temporal oblique line), governor vessel acupoints (e.g., GV20-*Baihui*, GV16-*Fengfu*, GV24-*Shenting*) and *Jiaji* acupoints (Ex-B2), or act directly on the spastic muscles (13–17). A very new systematic review that involves 88 RCTs with 6,431 patients in total has further confirmed that acupuncture possesses a reliable antispastic effect for stroke patients and summarized the most frequently used acupoints: LI4-*Hegu*, LI15-*Jianyu*, LI11-*Quchi*, SJ5-*Waiguan*, LI10-*Shousanli,* LU5-*Chize* and PC6-*Neiguan* for the upper limb, and GB34-*Yanglingquan*, ST36-*Zusanli*, SP6-*Sanyinjiao*, LR3-*Taichong*, SP10-*Xuehai*, SP9-*Yinlingquan* and ST41-*Jiexi* for the lower limb, which all locate at the muscle surrounding the affected joints (18). In comparison, research on antispastic acupuncture techniques is insufficient. In fact, specific acupuncture techniques applied on the acupoints are crucial for treatment outcome, which could be traced back to The Yellow Emperor’s Inner Classic (19). Nowadays, the perception of applying suitable acupuncture technique for relevant diseases is widely accepted in the clinical settings by more acupuncturists than ever worldwide (20–22).

As a major motor impairment following stroke, spasticity usually leads to structural and functional changes in skeletal muscles such as shortening and stiffness of muscle fibers, and discordant relationship between the flexor and extensor muscles, which in turn exacerbates motor dysfunction (23). Thus, the aim of PSS treatment should take into consideration not only alleviation of the disordered muscle itself but its impact on motor function. Motion-style acupuncture (MSA) is a distinctive branch of multiple acupuncture techniques. It belongs to the compound acupuncture technique, which is characterized by active or passive body movement (motor training) during needle retention (24, 25). Thus, MSA is not equal to conventional acupuncture plus motor training. It has been recommended for treating muscle and tendon disorders, such as spasticity, weakness, muscle soreness and restricted joint movement (26). Although an increasing number of clinical studies have indicated that the overall therapeutic effects of commonly used MSA techniques including waggle needling, *Dongqi* needling (DN), Fu’s subcutaneous needling (FSN) and motion-style scalp acupuncture (MSSA) are superior to conventional acupuncture/motor training alone, or their simple combination (27–35), few studies tried to unmask the possible mechanisms behind them. Based on the pathogenesis of PSS, combined with the characteristic of MSA techniques, certain new insights into acupuncture techniques for PSS in light of central and peripheral aspects will be discussed, with the purpose of optimizing antispastic acupuncture regimen and thereby helping with the improvement of patient’s quality of life.

## Understanding the plausible pathophysiology of PSS

In human, three important descending pathways are closely related to motor system, including CST originated from the cerebral cortex, and reticulospinal tract (RST) and vestibulospinal tract (VST) from the brain stem (36). Selective lesions to the CST only produce negative upper motor neuron (UMN) signs, such as weakness, hypotonia, and hyporeflexia, whereas the other two would cause positive consequences (e.g., spasticity) (6, 37). Although the pathogenesis of PSS has not been fully understood, mounting evidences indicate that spasticity is a result of changes in supraspinal origin, intraspinal network and peripheral muscle, leading to hyperexcitable stretch reflex (6).

First of all, supraspinal origin provides a balanced descending regulation for spinal stretch reflex circuit, which is predominantly mediated by RST and VST (38, 39). Specifically, the dorsal RST, receiving facilitation from the premotor cortex (PMC) and supplementary motor area (SMA) *via* corticoreticular pathway (CRP), descends paralleling with CST in the dorsolateral funiculus and wields a powerful inhibitory effect on stretch reflex (40). Conversely, the medial RST and VST, being independent from the contralateral motor cortex, in particular the anterior limb of the internal capsule with fibers from PMC, descend in the ventromedial cord and provide an excitatory effect (5, 41). Nevertheless, the contribution degree of VST to spasticity leaves a question open. It was shown that cutting off VST only caused transient alleviation of extensor tone of lower extremity in the spastic cat, however, spasticity markedly eased when the medial RST was broken (42). In stroke with cortical lesions, the medullary reticular inhibitory center, to a great degree, loses facilitatory inputs from the cortex and presents as weakened descending inhibitory effect. The medial RST, at this time, is in predominant state resulting in hyperexcitability of spinal stretch arc (5, 43).

Second, α-motoneuron (α-MN) hyperexcitability is deemed the abnormal intraspinal change in PSS (44). The reason is probably as follows: augmented sensitivity spindles increase peripheral afferent input to the spinal MN, setting the condition for oversensitivity of the stretch arc (39). Moreover, changes in intrinsic properties of the spinal MN lead to weakened presynaptic and reciprocal inhibition that enable the MN to spontaneously discharge, and ultimately decrease reflex threshold (39, 45). It is likely that resultant α-MN hyperexcitability is a plastic reorganization following lopsided supraspinal descending inputs to the spinal network (6). Bernice et al. has found that within limited temporal window poststroke, both brain and spinal cord initiate enhanced structural reorganization depending on the degree of cortical insult, which further promotes functional recovery (46).

Likewise, levels or expressions of certain neurotransmitters in the central nervous system (CNS) have long been elucidated crucial in the pathophysiology of spasticity as well. These neurotransmitters could be basically divided into two categories: one is inhibitory neurotransmitters including γ-aminobutyric acid (GABA) and glycine (Gly), the other excitatory ones like glutamate (Glu) and aspartate (Asp) (47). Normally, neurotransmitters bond to their receptors to modulate monosynaptic and poly synaptic reflexes in spinal level and mutually maintain the balance between excitation and inhibition of neurons (48, 49). However, once the balance is disrupted, i.e., either increased release of excitatory neurotransmitters or lessened activity of inhibitory inputs, spasticity occurs or aggravates (47, 48, 50). In addition, few studies reported that the inflammatory response is also involved in the pathogenesis behind PSS that we will discuss later (51, 52).

**Figure 1:**
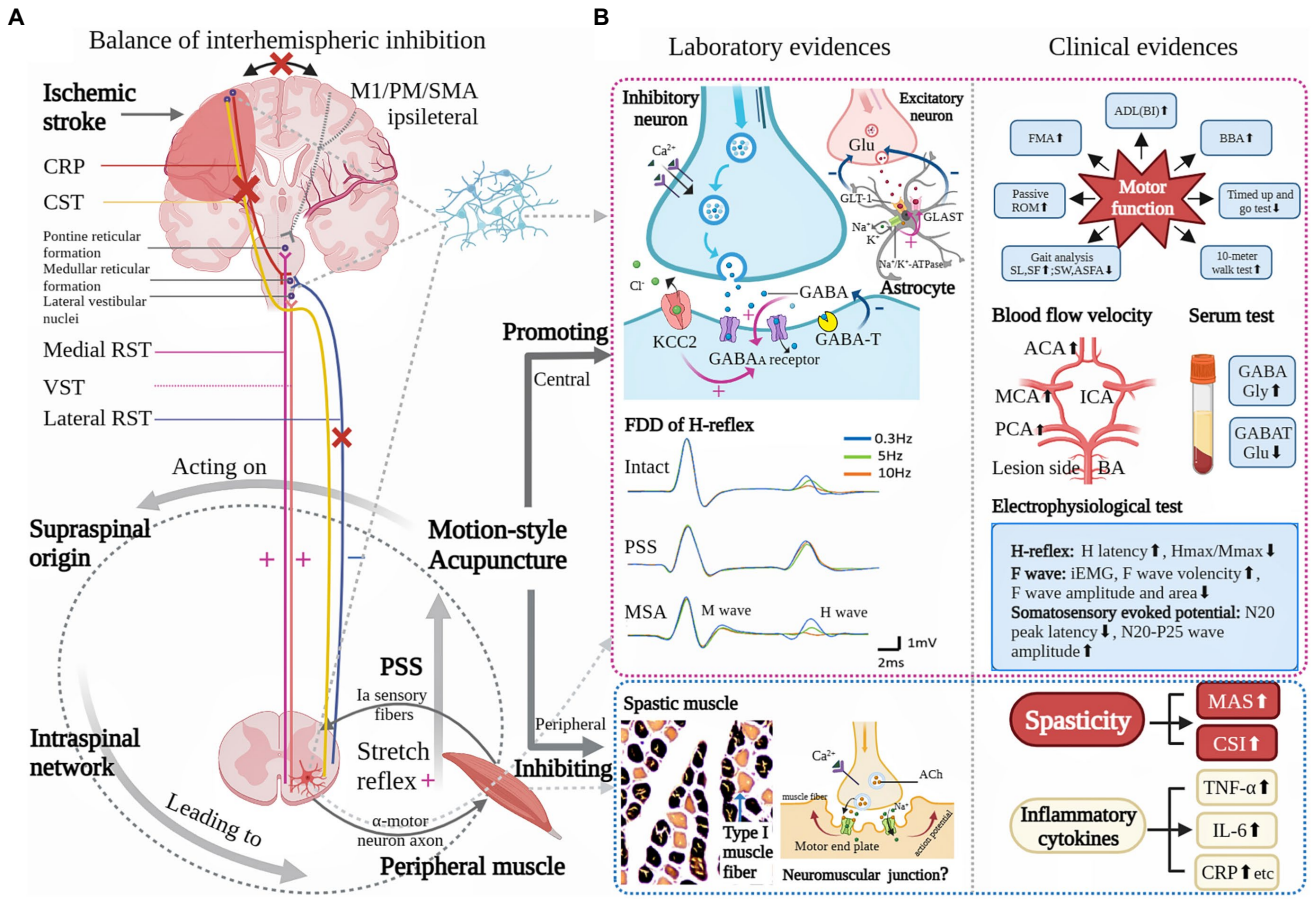
Underlying pathophysiology of poststroke spasticity (PSS) and role of motion-style acupuncture (MSA) for PSS. **(A)** is modified from Li et al. (2) showing that the possible primary mechanism for a loss of inhibitory control after a stroke is an UMN lesion while the secondary factors are altered intraspinal network processing and peripheral muscular changes. **(B)** Based on current clinical and laboratory evidences, the possible role of MSA for PSS is mainly reflected in two aspects: one is its modulation on the CNS, the other peripheral muscle. Central modulation presents relative neuroprotective effect (improvement of blood perfusion and inhibition of inflammatory response), regulation of balance between excitatory and inhibitory neurotransmitters, functional improvement of sensorimotor pathway, and inhibition of spinal stretch reflex. Peripheral muscle adjustment includes decreased proportion of type 1 muscle fiber. ACA: anterior cerebral artery, Ach: acetylcholine, ADL: activity of daily life, ASFA: affected side foot angle, BA: basilar artery, BBA: Brunel balance assessment, BI: Barthel Index, CRP: C-reactive protein, CSI: clinical spasticity index, CST: cerebral-spinal tract, FDD: frequency-dependent depression, FMA: Fugl-Meyer assessment, GABA: γ-aminobutyric acid, GABAA: γ-aminobutyric acid subtype A, GABAT: γ-aminobutyric acid transaminase, GLAST: glutamate aspartate transporter, GLT-1: Glutamate transporter-1, Glu: glutamate, Gly: glycine, ICA: internal cerebral artery, iEMG: integrated electromyography, IL-6: interleukin-6, KCC2: K(+)-Cl(−) co-transporter, M1: primary motor cortex, MAS: modified Ashworth scale, MCA: middle cerebral artery, PCA: posterior cerebral artery, PM: premotor cortex, ROM: range of motion, RST: reticulospinal tract, SF: slide frequency, SL: slide length, SMA: supplementary motor area, SW: slide width, TNF-α: Tumor necrosis factor-α, VST: vestibular spinal tract. (+): excitatory, (−): inhibitory, (×): impaired, (⬆): increased, (⬇): decreased.

Last but not least, it must be stressed that PSS is a complex clinical phenomenon, which is not only considered as a neurologic problem but an indication of muscle property alteration. The alteration, for example, includes increased proportion of connective tissues, decreased amount and shortened length of sarcomeres (41). When a paralyzed muscle is held in an abnormally shortened position, it would lose sarcomeres to adjust its length and thus potentiate contracture, which in turn aggravates spasticity (5). Besides, researchers found that there is a strong connection between increased proportion of type I muscle fibers and muscle hypertonia in spasticity patients (53). To sum up, hyperexcitability of the medial RST tends to be the key mechanism, while changes in intraspinal network processing and peripheral muscle property are secondary factors that contribute to the development of PSS (5, 6, 43; Figure 1A).

## Characteristics of commonly used MSA techniques

As mentioned before, waggle needling, DN, FSN as well as MSSA all highlight effective integration of acupuncture technique and rehabilitation, that is cooperating with motor training during needle retention. Because of this, MSA treatment not only promotes positive interaction between acupuncturists and patients, but creates conditions for those with neuromuscular retraining, which helps to improve patient compliance and treatment outcomes (24, 26). Nevertheless, each of MSA techniques has its own characteristic. For example, as for needling sensation, waggle needling, DN and MSSA are supposed to evoke relatively strong yet tolerable needling sensation; however, not in FSN. The principle of acupoint selection varies among them as well, which could be preliminarily classified as local and remote point selection. Waggle needling and FSN are always applied on the points in the affected areas, e.g., some classic acupoints closely related to tendons (like GB34, SP9, ST41, LI11 and LI15) (28) and the most significant tender point (positive reaction point) (54–56). However, the exact point of needle insertion is quite different. In waggle needling, the tender point should be needled at its exact location, while in FSN, 2–3 *cun* (1 cun = 3.33 cm) superior or inferior towards it. Given that different needling directions towards the tender point would not influence therapeutic effects of FSN (57), thus, the point selection of FSN is more flexible and adjustable than the waggle needling. In contrast, the stimulating points of DN and MSSA are far away from the affected muscles and tendons, named remote point selection. The primary distal acupoints (*Tung’s* extraordinary points) on the healthy side (58) and specific areas or lines (MS6, MS7) on the scalp (59) are the main needling target for DN and MSSA respectively, which differs considerably from the traditional acupuncture theory. It has to point out that long-durational needle retention can be realized in both MSSA and FSN, particularly the latter, for up to 24–48 h, attributing to superficial needling depth that has no impact on deep muscle layers and limb exercise, which may increase the total amount of stimulation and contribute to long-term therapeutic effect (32, 57). Detailed characteristics among these MSA techniques are summarized in Table 1.

**Table 1:** Characteristics among the commonly used MSA techniques.

References	Similarities				Differences			
	Style of acupuncture	Clinical indications	Instrument	Area of needle stimulation	Principle of acupoint-selection: the main points	Needling sensation	Long needle-retention	Manipulation characteristics
Waggle Needling (28, 29, 60)	Motion-style acupuncture is unique in that effective and synchronous combination of acupuncture with body movement. Simultaneous activation of sensory and motor conduction pathways favors reconstruction of the right neural feedback mechanism and improvement of motor ability	Neurological and muscular diseases (e.g., spastic hypertonia, contracture, joint immobilization, muscle stiffness and pain)	Filiform needle	A greatest area with muscle tissues at multiple layers in a three - dimensional area around the stimulated point	Local point-selection: Points around the joint (e.g., LI15, LI11, SJ5, LI14 for the upper limb; GB34, SP9, SP10, ST36, ST41, SP6 for low limb) or the tender point, mainly on the affected side	Strong yet tolerable	No (For about 30 min)	Penetrating from the skin to the muscle layers followed by lifting-thrusting methods in multi-directional angles
Fu’s subcutaneous needling (61–63)			Fu’s needle	A greater area with subcutaneous tissues in a fan-shaped area outwards from the inserting point	Local point-selection: 2–3 *cun* superior or inferior to the most significant tender point, mainly on the affected side	None-requirement	Yes (Up to 24–48 h)	Puncturing subcutaneously followed by swaying method, with a long-period of soft tube retention
*Dongqi* needling (30, 31, 58)			Filiform needle	A limited area with the muscle tissues in the longitudinal area at the stimulated points and its deep tissues	Remote point-selection: *Tung’s* extraordinary acupoints [e.g., T 77.18 (bilaterally) and T 22.05, T 22.04, T 77.07 on heathy side]	Strong yet tolerable	No (For about 30 min)	Penetrating from the skin to the muscle layers with twirling or lifting-thrusting methods
Motion-style scalp acupuncture (64–66)			Filiform needle	A limited area with the tissue in the transverse area at the stimulated points and its relatively shallow tissues	Remote point-selection: the anterior (MS6) and posterior (MS7) oblique lines of the vertex-temporal on the affected side	Strong yet tolerable	No (For about 30 min)	Penetrating from the skin to the epicranial aponeurosis followed by quick twirling method for about 200 rpm/min

## Evidences from clinical studies

### MSA techniques alleviate muscle tone and improve motor functions in PSS patients

In the real clinical setting, except for spasticity, stroke patients may suffer from additional complications, for instance, motor dysfunctions manifesting as low task-oriented executive ability, joint immobilization, abnormal posture and spastic muscle pain. Therefore, employing combined approaches rather than conventional monotherapy to counteract those complex conditions has aroused wide attention among doctors and researchers. Loads of clinical studies have shown that the antispastic effect of MSA is superior to routine needling or rehabilitation alone. In one RCT, 121 patients with PSS were randomly sorted into control group by routine needling and treatment group by waggle needling. All these patients were given similar usual care, including neurotrophic supplement, anti-infection, blood pressure control, microcirculation improvement, and so on. Although both groups showed positive effects in spastic hemiplegia after 2-weeks of intervention, shown as decreased modified Ashworth scale (MAS, the most commonly used scale for spasticity assessment) score but increased Fugl-Meyer assessment (FMA, to evaluate overall motor function) and Barthel Index (BI, to evaluate activities of daily living) score, inter group comparison displayed that the overall effect of waggle needling is better than routine needling (*p* < 0.05) (28). Another study (34) conducted a RCT on 140 PSS patients, comparing MSSA treatment (*n* = 70) with intelligent upper-limb feedback training alone (*n* = 70). The results showed that after 8-weeks treatment, the spasticity degree of the elbow (69.07 ± 9.39) and wrist (33.04 ± 7.33) joints in the MSSA group were significantly lower than that in the rehabilitation training group (80.65 ± 7.98 and 35.91 ± 7.50, respectively). Besides, the overall treatment outcomes (MAS, FMA, BI) and long-term efficacy of MSSA is much superior to rehabilitation training alone after 1-month follow-up. Ge et al. (58) observed similar outcomes. Sixty PSS patients were randomly assigned into DN group (*n* = 30) and routine needling group (*n* = 30). The MAS, FMA, BI, balance function (Brunel balance assessment, BBA), walking ability including timed up and go test (TUG) and 10-meters walking speed test, and stroke specific quality of life scale before and 4-weeks after intervention were measured. The results showed that while the above outcome measures significantly improved in both groups (*p* < 0.05), the improvements of motor function of lower limbs in DN group (BBA: 40.19 ± 5.21, TUG: 21.77 ± 2.65, 10-meters walking speed: 48.22 ± 5.37) are better than those in the routine needling group (27.56 ± 2.73, 28.91 ± 3.50, 39.05 ± 4.53, respectively) (all *p* < 0.01). On top of its remarkable effects on meaningful reduction in the degree of spasticity with improvements in motor function and quality of life in patients, FSN owns superiority in immediate pain relieving in comparison with other three MSA techniques, possibly thanks to its effect on directly loosening tissue adhesion by swaying manipulation (32, 55, 61).

Recently, a meta-analysis involving 31 RCTs with total 2,488 PSS patients indicated that a range of outcome measures including marked efficiency, MAS classification, MAS score, and clinical spasticity index (CSI) score, in electroacupuncture plus rehabilitation training group were all statistically better than those in electroacupuncture or rehabilitation training alone groups (67), inspiring us that combination of different therapies (e.g., MSA plus professional rehabilitation training) may maximize spasticity management. Song et al. (62) observed the effect of combination therapy versus monotherapy. One hundred and twenty PSS patients were randomly distributed to rehabilitation group (*n* = 40), FSN group (*n* = 40) and FSN plus rehabilitation group (*n* = 40). The final outcomes showed that the muscle tone (MAS), hand motor function (FMA) and range of motion (ROM) of wrist joint were all significantly improved among the three groups (*p* < 0.05), however, the overall effects of combination group are far better than those of FSN or rehabilitation alone group (*p* < 0.05). No statistical differences existed between the FSN and rehabilitation group (*p* > 0.05). Similar outcomes were also shown in other two studies that antispastic effect of combination group (DN + rehabilitation) is superior to DN treatment or rehabilitation alone (30, 31). In addition, spasticity is reported to become worse in cold (6), which is consistent with the TCM theory that cold pathogen could contract muscles, obstruct meridians and thereby aggravate symptoms. Wang et al. observed therapeutic effects of combination therapy (waggle needling with moxibustion) for PSS. The results showed that the combination group presented higher integrated electromyography (iEMG) values and F wave velocity in both biceps and triceps, but lower F wave amplitude and area than the waggle needling group, indicating alleviation of spasticity and increasement of muscle strength (56). This study enlightens us that keeping warm during MSA intervention may help relax muscles of patients easily affected by cold temperature and achieve better outcomes.

To date, mounting studies have indicated that the order of treatment or timing of combination of different therapies, such as synchronous or asynchronous application of acupuncture and rehabilitation, may bring different prognostic outcomes to patients (35, 64, 65). For instance, a RCT by Qi et al. (68) has shown that after 6-months treatment, muscle hypertonia and motor functions were all substantially improved among three treatment groups (rehabilitation alone, rehabilitation after scalp acupuncture and MSSA groups) in comparison with before treatment. And compared to the other two groups, MSSA possessed preferable therapeutic effects (*p* < 0.05). Zhang et al. (33) found that the therapeutic effect of conventional scalp acupuncture plus rehabilitation is better than scalp acupuncture alone, but inferior to synchronous combination of scalp acupuncture and rehabilitation (MSSA). The efficacy is reflected in ameliorating patients’ gait parameters, including increased stride frequency and stride length and lowered foot angle of the affected side, and increased passive ROM of joints (the hip, knee and ankle). Some researchers held the opinion that in the process of neuroplasticity, active and proper rehabilitation inducement is particularly essential to avoid establishment of abnormal movement pattern and unexpected motor compensation. In MSA, the sensory and motor conduction pathways can be activated simultaneously, owning to the dual of acupuncture manipulation and active/passive body movement, which conduces to restoration of right neural feedback mechanism and reconstruction of motor ability (34, 66, 68). Taken together, results from these clinical trials (although still limited) imply that MSA is a promising and effective therapy for patients with PSS.

## Evidences from animal studies

### MSA techniques impact on behavioral performances in rats with PSS

In contrast to the wealth of clinical studies, comparatively fewer experiments exist exploring the effect of and underlying mechanism of MSA techniques against PSS in rodent animals. Currently, the most popular PSS model of rats is established through middle cerebral artery occlusion (MCAO) surgery, featuring by high reliability and good reproducibility (69). Our previous studies employed Zea Longa score and MAS to evaluate neurological deficit and muscle tone in the model rats, separately (70–73). At the very beginning, all rats scored 0 in Zea Longa and MAS before MCAO, indicating there is no neurological deficit and no spastic hypertonia. Three days after modeling, Zea Longa score was still 0 in the normal and sham-operated groups. Meanwhile, the score in other modeled groups (model, waggle needling, routine needling, and Baclofen groups) were all statistically higher than the sham-operated group (all *p* < 0.01), indicating the neurological deficit was successfully induced. After 7-consecutive-days intervention, Zea Longa score has obviously decreased in the three intervention groups (*p* < 0.01 or *p* < 0.05) in comparison with the model group. And waggle needling showed similar therapeutic effect on alleviation of neurological deficit as Baclofen does (*p* > 0.05). The MAS score normally increases 3 days after MCAO, maintains at a high level within 9 days, and decreases gradually later, which has been proved by previous animal models (69). In order to exclude the influence of animal self-recovery on the experimental outcomes, we started the intervention at day three after modeling and terminated the experiment at day nine. The results showed that acupuncture, in particular waggle needling, significantly decreased MAS score when compared to the model group (*p* < 0.05). These data confirmed that waggle needling wields preferable effects on neurological deficit and spastic hypertonia. As for the underlying mechanisms, they are possibly related to microcirculation improvement of peri-infarct areas, inhibition of inflammatory responses, and alterations of neurotransmitters, spinal reflex as well as muscle property, which will be discussed in the following part.

In addition, efforts have been made to quantify the behavioral deficits in animals with some indirect assessments like screen test and gait analysis. We found that rats suffered from motor dysfunctions immediately after MCAO and manifested with weakness and imbalance, which might be caused by damage to the cerebral cortex and CST. The screen test scored five in the sham-operated group but only about two in the modeled groups 1 day following surgery. With acupuncture intervention, motor function was obviously elevated at day seven and day nine in the waggle needling group, compared with the model group (all *p* < 0.01). No statistical differences existed between routine needling and model groups (*p* > 0.05) (71). An experiment conducted by Mu et al. (74) observed hindlimb balance and state of gait movement in rats with PSS. The PH-200 ft. balance tester showed that rats in the sham-operated group showed no changes in the static weight ratio throughout the experiment, however, the ratio in model, waggle needling and placebo needling groups obviously decreased on the day three after MCAO. Following 7-treatment-days, the ratio was effectively augmented by waggle needling compared to the model group (*p* < 0.001). Likewise, before treatment, significant enlargements of swing, stance, step angle and stance width but reductions of peak paw area and stride length were shown in the model, waggle needling and placebo needling groups when compared to the sham-operated (all *p* < 0.01). Interestingly, this situation was significantly reversed by waggle needling after treatment, indicating that waggle needling possesses the ability to facilitate motor function recovery. Recently, a study has proved that spastic behavior of rat was consistent and reproducible during swimming test (75), such a behavioral test is desired to bring more convinced evidences to the antispastic effect of MSA techniques.

## Modern insights of MSA techniques for PSS

### Inspiration for MSA techniques in light of facilitating central neuroplasticity and neuromodulation

Neuroplasticity is defined as the ability of neurons and circuits to modify their functional activity and the synaptic reconstruction in accordance with variations in activity (16). Enhancement of neuroplastic activity generally exerts a positive effect on motor recovery. After a stroke, neuroplasticity initiates immediately in bilateral cortices, which could be explained by structural change and regeneration, cortical reorganization (neural excitability, CST integrity and intracortical excitability) and molecular biology changes (76–79).

Impaired nerve cells in the penumbra surrounding the ischemic core could be saved only if the blood perfusion is timely restored (80). Ischemic insult triggers angiogenesis to support penumbra, and induces neurogenesis and synaptogenesis in the perilesional cortex (81, 82). Restoration of blood perfusion to the ischemic areas was therefore considered as a vital therapeutic target for rehabilitation outcomes (16). Both clinical and experimental studies have proved the satisfying effects of MSA techniques (DN, waggle needling) on microcirculation improvements, including promoting mean blood flow velocity of posterior cerebral artery (PCA), middle cerebral artery (MCA) as well as anterior cerebral artery (ACA) in patients with PSS (58), and reducing cerebral infarct volumes in the model rats (70–72, 74). Additionally, there is a study indicating that inflammatory response during ischemic process can induce a cascade of negative consequences, for instance, promotion of leukocyte infiltration and obstruction of capillary blood flow, which in turn aggravates the extent of ischemic areas and cell necrosis (52). Early acupuncture intervention, in particular MSA (waggle needling), could effectively inhibit the expression of inflammatory markers (e.g., Tumor necrosis factor-α, interleukin-6 and C-reactive protein) in the serum in patients with PSS, which favors the improvement of regeneration and functional recovery of CNS (52, 60). These outcomes can mean that MSA possesses relative neuroprotective effects on ischemic stroke. Relevant studies have declared that certain neuroprotective factors such as brain-derived neurotrophic factor, postsynaptic density protein 95, synaptophysin favor neurogenesis, synaptic plasticity (83); and the active neural plasticity in the perilesional cortex may create opportunity for new synaptic connection (e.g., CRP), which contributes to regaining the facilitation from the contralateral PMC to the medullar inhibitory center and thereby restores downward inhibition to the stretch reflex.

It was reported that activation of contralateral hemisphere is greater than that of the ipsilateral in the subacute stage so as to compensate for severely damaged motor control; while the activation will shift back to the ipsilateral that facilitates recovery of voluntary control on the paretic side in the chronic stage (79, 84, 85). For treating stroke patients suffering from motor deficits, scalp acupuncture could markedly activate motor cortex, strengthen the activities of the brain regions related to sensory integration and motor coordination, enhance bilateral frontal lobe motor control and thereby conduce to the cooperation of bilateral sensorimotor networks and the balance between inter-hemisphere (86–89). After stroke, mountains of neurons and nerve fibers are damaged, directly causing sensory and motor deficits with abnormal somatosensory evoked potential (SEP) responses that mainly presents prolonged relative peak latency and lowered wave amplitude. Interestingly, recent studies have indicated that the N20 peak latency of the median and tibial nerves were obviously shortened, while the N20-P25 wave amplitude of these two nerves were increased in MSA group (motion-style scalp acupuncture) as compared to conventional scalp acupuncture plus rehabilitation group or scalp acupuncture group (64, 66).

Disequilibrium of neurotransmitter adjustment such as overexpression of excitatory neurotransmitter (like Glu) and/or insufficiency of inhibitory neurotransmitters (GABA, Gly) was found in the biomolecular mechanisms underlying PSS in both human and animal studies. Clinical results showed that in comparison with routine needling alone, spastic hypertonia and motor dysfunction were significantly alleviated in MSA (DN, MSSA or waggle needling) group, accompanying by suppression of Glu, and elevations of GABA and Gly in serum in patients with PSS (58, 60, 66). Animal studies also confirmed that the antispastic effect of waggle needling was superior to routine needling and equivalent to Baclofen (70–73). Underlying mechanisms could be explained as follows: In the perilesional cortex, the expression/activity of GABA was enhanced but GABAT (a key metabolism enzyme of GABA) was inhibited. In addition, expressions of GABA_A_γ2 receptor and KCC2 (a chloride extruder that maintains the inhibitory effect of GABA) were all elevated among the cerebral cortex, brain stem and lumber spine of PSS rats. Furthermore, downregulations of Na^+^/K^+^-ATPase and Glu transporters (EAATs) such as EAAT1 (GLAST) and EAAT2 (GLT-1), and upregulation of Glu in the hippocampus were effectively reversed by waggle needling. Such experiments are needed to help us understand the biochemical mechanisms behind MSA techniques for PSS.

### MSA techniques contribute to the improvement of peripheral muscular conditions

The possible primary mechanism for a loss of inhibitory control after a stroke is an UMN lesion while the secondary factors are altered intraspinal network processing and peripheral muscular changes, which can both result in PSS (6). These imbalanced inputs from the supraspinal levels partly lead to α-MN hyperexcitability, subthreshold or spontaneous discharging of motor units, associated with spastic hypertonia and muscle contraction (6). Moreover, peripheral muscular conditions have changed as well, including muscle fiber shortening and stiffness, loss of sarcomeres in series, connective tissue adhesion, and increased proportion of type I muscle fibers, which additionally exacerbate joint immobilization and motor dysfunction in patients (53, 90–92). However, those conditions could be soothed with prolonged slow passive stretching, to prevent excessive muscle contracture and lessen spasticity-induced pain (10, 93).

According to the classical acupuncture theory, therapeutic effects of acupuncture are determined by acquired needling sensation (*deqi* in Chinese) (94, 95), and different types of stimulation normally bring about diverse clinical outcomes (96). It was shown that stroke patients suffering from spastic hemiplegia react especially well to a type of acupuncture with strong needle-stimulation (97). Effective needling manipulation, particularly like multi-directional lifting-thrusting method in waggle needling, or swaying method in FSN, enables increased changes in local blood perfusion and soft-tissue displacement, delivering mechanical signals into the subcutaneous tissue, inducing the release of pain-related substances and consequently triggering the nerve-immune-secretion network to relieve spastic muscle pain (94, 98). Modern research shows that immediate alleviation of spasticity and increase of active ROM in PSS patients are positively influenced by quick lifting-thrusting needling method at trigger point, which correlated with decreased frequency of motor unit spontaneous discharging (99). H-reflex is a monosynaptic reflex triggered by activation of Ia afferents, MN and muscle fibers, and is extensively used as a valid tool to quantify the excitability of the MN (100). Certain parameters of H-reflex changed in patients with PSS, such as shortened H-reflex latency, amplified Hmax/Mmax ratio. Animal research also demonstrated that motor threshold, frequency-dependent depression (FDD) of H-reflex were all increased, indicating that occurrences of hyperreflexia and spastic hypertonia correlated with hyperexcitability of MN. While those conditions could be effectively reversed by MSA techniques (63, 74). Besides, the proportion of type I muscle fibers in the spastic gastrocnemius muscle is markedly declined after waggle needling intervention in comparison with that of routine needling group (*p* < 0.05) (72). As reported, the direct action of lifting-thrusting needling method on peripheral muscular tissues (e.g., neuromuscular junction and muscle fibers) possibly correlates with breaking down of muscle fibers and temporary depletion of acetylcholine neurotransmitters (101). Whether those mechanisms are involved in the antispastic effect of MSA (especially waggle needling and FSN that directly act on spastic muscles) remains to be further investigated. To sum up, to improve the intrinsic structure of spastic muscles and to attenuate over-excitation of the spinal reflex circuit may serve as meaningful therapeutic targets for MSA against PSS. Such electrophysiological studies will help to provide more objective evidences in showing the effects of different MSA techniques on peripheral muscular conditions. Relevant evidences are presented in Figure 1B.

## Discussion and expectation

Given that both clinical and experimental evidences (although still limited) has confirmed the preferable antispastic effect of MSA techniques, also seeing that at present there still lacks standardized regimen for PSS management, MSA deserves to be considered as a part of comprehensive therapeutic protocol for stroke patients who suffer from spasticity, with its merits of preferable effect, few acupoint-selection as well as affordable price, and importantly, attributable to its potentially regulatory effects on the CNS and peripheral muscles. However, it has to be mentioned that MSA techniques involved in previous studies are normally based on TCM theory or personal experiences. In views of TCM, obstruction of meridians, *qi* and blood stagnation, or poor nourishment of muscles and tendons after stroke consequently led to edema, pain, numbness, stiffness and disadvantageous movement of distal limbs. Intensive amount of needle stimulation (e.g., relatively strong but tolerable stimulation intensity, large stimulation area or relatively long-durational needle retention) integrated with tailored motor training determines that MSA plays a meaningful role in accelerating the flow of *qi* and blood, unblocking meridians and easing muscles in PSS patients (50). However, the intervention timing and course, acupoint-selection, and exercise mode are different among them, limiting the curative efficacy and credibility of MSA studies to some extent. To further optimize treatment protocol of MSA for PSS, certain insights based on the practice implications are provided from the following aspects.

### Intervention timing and course

Proper intervention within optimal time window could facilitate neuroplasticity, and to a great extent, avoid maladaptive neuroplasticity (spasticity). Neuroplasticity begins within hours after ischemia, peaks at 7–14 days poststroke in rodent animals (102), while it peaks within 3 months poststroke in human beings (79). It has been found that in animals, a very early exercise within 24 h poststroke results in overexpressed inflammatory responses (103). Conversely, rehabilitation commencing later (24–72 h poststroke), is favorable to suppressions of ischemic volumes (82), inflammations (104) and apoptosis (105), yet promotion of neurogenesis (83). Similar outcomes are observed in human beings that exercise less than 24 h after stroke is related to infarct expansion (106), but within 72 h is considered beneficial (107). Considering that early acupuncture intervention could induce significant ischemia tolerance (108, 109), and the intensity of motor training (active/passive body movement-based) in MSA is relatively moderate, on the basis of internal medicine care that helps to open the obstructed blood vessels timely (e.g., application of recombinant tissue-type plasminogen activator, t-PA) (110), it might be a feasible choice to apply MSA as early as possible (after hyperacute phase, ≥24 h), and to insist on it at least 2 to 3 months as most of previous MSA studies did (33, 34, 54, 65, 66), for stroke patients to actively combat ischemic injury and facilitate neuroplasticity.

### Selection and application of MSA techniques

Previous research has demonstrated that pathogenesis of spasticity in the early subacute phase (> 7 days–< 3 months) after stroke could be explained by neural changes; whereas, as time goes by, during the late subacute to chronic stages (≥3 months-2 years), it might link with intrinsic muscle alterations (4). A factorial study supported the view that both “needling stimulation” and “acupoint selection” contribute to the acupuncture efficacy (97). Although there is insufficient scientific evidence of high-quality suggesting that needling technique wields more impacts on the clinical result, some experts have argued that it is more important to perform suitable acupuncture techniques than to select exactly the right acupoint with routine needling technique (111). Therefore, the choice of needling technique should also be flexible for spasticity treatment at different poststroke phases. In light of previous evidences from the clinic to the laboratory, also taking needling features of MSA techniques into consideration, an auxiliary MSA treatment protocol for PSS is designed as follows.

Early after a stroke (in the hyperacute and acute phases), before spasticity forms, treatment should focus mainly on promoting neuroplasticity and avoiding maladaptive neural reorganization. Given that scalp acupuncture tends to enhance cerebral blood reperfusion and facilitate integration and cooperation of sensorimotor cortex network (86, 112, 113), and also considering that the contralateral needling (in healthy side) in DN technique might activate the uncrossed CST to compensate for contralateral CST, which is based on symmetry constraint (114), MSSA and DN techniques are therefore recommended in the early poststroke stage, in light of facilitating neuroplasticity and neuromodulation. Over time, stroke patients, without effective intervention, would undergo a series of motor impairments, such as synergic movement pattern and different degrees of spasticity. At this time, not only the CNS lesions should be considered, but the changes in the intrinsic muscle properties should be paid attention to. As connective tissue adhesion and increased proportion of type I muscle fibers potentiate the degree of spastic hypertonia (53, 92), applying waggle needling at disordered muscles (the tender point or tendon-related acupoints) with multi-directional lifting-thrusting method can be regarded as a good way to sooth spastic muscle and improve its structure (28, 29). Besides, except for directly acting on local areas as waggle needling does, FSN possesses the ability in immediate pain-relieving by loosening connective tissue adhesions with swaying method (54, 57, 61), which ultimately helps with restoration of mechanical alignment and improvement of motor performance.

Moreover, it is noteworthy that both hemodynamics and electrophysiology studies support the view that stroke patients who have received bilateral needling showed preferable effects as compared to unilateral needling (115–117). After treatment, the blood velocity of ACA, MCA, PCA or basilar artery was all strengthened among the unilateral needling, bilateral needling and conventional medicine groups (*p* < 0.01 or *p* < 0.05). Most importantly, the blood velocity of ACA, MCA and basilar artery (contralateral side) in the bilateral needling group was superior to unilateral needling group (*p* < 0.05). Likewise, bilateral needling group had more advantages in the improvement of SEP responses (e.g., reduction of latency, increasement of amplitude, reproduction of missing amplitude), hinting that neurons and fibers recruited during bioelectrical activity were increased, and so was bilateral cortical excitability to some extent. In addition, rehabilitation research demonstrated that bilateral hands rehabilitation led to decreased intracortical inhibition yet increased intracortical facilitation in stroke patients, while these alterations only occurred in the contralateral hemisphere in patients with unilateral hand training (118, 119). In line with these mechanisms of improvements after stroke, applying MSA bilaterally might be a noteworthy insight due to its better effects on enhancing neuroprotection and rebalancing of interhemispheric inhibition.

### Learning from and integrating with modern rehabilitation strategy

In view of the fact that the exercise mode in most of MSA techniques is active/passive movement of the disordered limb, lacking of standardized rehabilitation strategy, to some extent, limits curative efficacy of MSA in real clinic settings. Because of the complexity and variability of PSS, apart from classic pharmacological modulations on CNS, advanced rehabilitation strategies targeting to improve peripheral muscle conditions (11, 120–122), for instance, biomechanical restoration *via* muscle stretching, improvement of motor control by body weight–supported treadmill training or robot-assisted training, enhancement of muscle strength by resistance training program or aquatic therapy, and improvement of endurance *via* treadmill exercise and circuit training, are worthy to be learned and implemented in the portion of motor training in MSA practice. Of course, the intensity of motor training should also depend on the patient’s condition. For patients with mild to moderate motor impairments, active motor trainings such as weight–supported treadmill training or task-oriented training are particularly recommended during MSSA and FSN treatments, because transverse needling could ensure the range of movement as large as possible. On the contrary, passive motor trainings, e.g., muscle stretching, robot-assisted training, proprioceptive neuromuscular facilitation, are more suitable for those with severe neurological impairments that cannot perform tasks by themselves, and can also be combined with MSA techniques flexibly. Notably, stroke patients tend to manifest less endurance with easy fatigue (2). Thus, the frequency and intensity of needling manipulation and motor training should not be in a state of excess, that is, avoidance of a single excessive stimulation to insure against hyperalgesia (26, 123). Instead, with the suggestion that repeated needling manipulation and motor training at proper intervals during needle retention is taken into consideration along with achieving proper stimulation intensity and tolerance of patients. For safety reason, the patient’s body position should be adjustable and comfortable throughout the whole treatment process to avoid acupuncture-related adverse-events such as bending or breaking of the needle, or fainting during treatment.

### Predictive and aggravating factors of spasticity

The aim of PSS treatment should take into consideration not only alleviation of spastic hypertonia but its underlying impact on motor rehabilitation. Early positive treatment may not terminate PSS progress, but may greatly reduce the incidence of severe spasticity and prevent intractable complications (8, 124). Certain factors are reckoned as important and independent predictors of PSS: lesion location (especially in the brain stem), degree of neurological impairments (severe paresis and hemihypesthesia, National Institutes of Health Stroke Scale >2, Mini-Mental State Examination <27), functional limitations (Modified Rankin Scale >2, low BI), large infarct volumes, increased MAS score, younger age and stroke-related pain (8, 125). Thus, to know these possible PSS predictors and to solve these problems pointedly with certain neuroprotectants, e.g., calcium channel blockers, Glu antagonist, free radical scavenger, or anti-inflammation drugs are advantageous to reduce the stress response under brain pathological conditions, inhibit inflammatory responses, and promote neural regeneration and neural repair in acute ischemic stroke, which may create positive prerequisites for MSA techniques to achieve the best therapeutic outcomes (126, 127).

Besides, clinical observations have also indicated that spasticity changes with posture, temperature weather (worse in cold), pain and emotion (anxiety, anger) (6). Tailored and systematic TCM nursing protocol, for example, normal limb placement plus exercise nursing, body warm-keeping with moxibustion or hot compress therapy, pain care as well as emotional nursing care (128), are especially recommended to deal with up-mentioned aggravating factors. Meanwhile, given that poor nutritional status is common among stroke survivors that not only leads to muscle loss but negatively impacts on poststroke recovery (129), positive solution to malnutrition is therefore considered as a meaningful supplement for the conventional treatment.

## Limitations and future research

Although acupuncture, in particular MSA techniques, is supported by a vast amount of clinical and experimental results in dealing with various motor impairments following stroke, however, there still exists certain limitations. Quite a few MSA studies are limited to relatively small sample sizes. In addition, other factors including methodological deficits in randomization, inconsistent assessment of spasticity (e.g., using different scales), subjective evaluations (clinical scale-based evaluations like MAS and FMA), incompatible inclusion/exclusion criteria (e.g., ischemic or hemorrhagic stroke types), differences in the time between stroke onset and assessment, differences in the intervention timing and course, acupoint-selection, comparatively simple exercise mode used in MSA techniques, absence of long-term follow-up, partly reduced the quality of the current MSA studies. Few studies were able to determine whether the observed effectiveness of MSA was due to placebo effects, the intensity of practitioner contact, or the physiologic effect of needling. In brief, although positive outcomes were found that the antispastic effects of MSA outdo conventional acupuncture alone, rigorous evidence-based meta-analysis is highly expected in the future, to provide acupuncturists with more powerful guidance.

**Figure 2:**
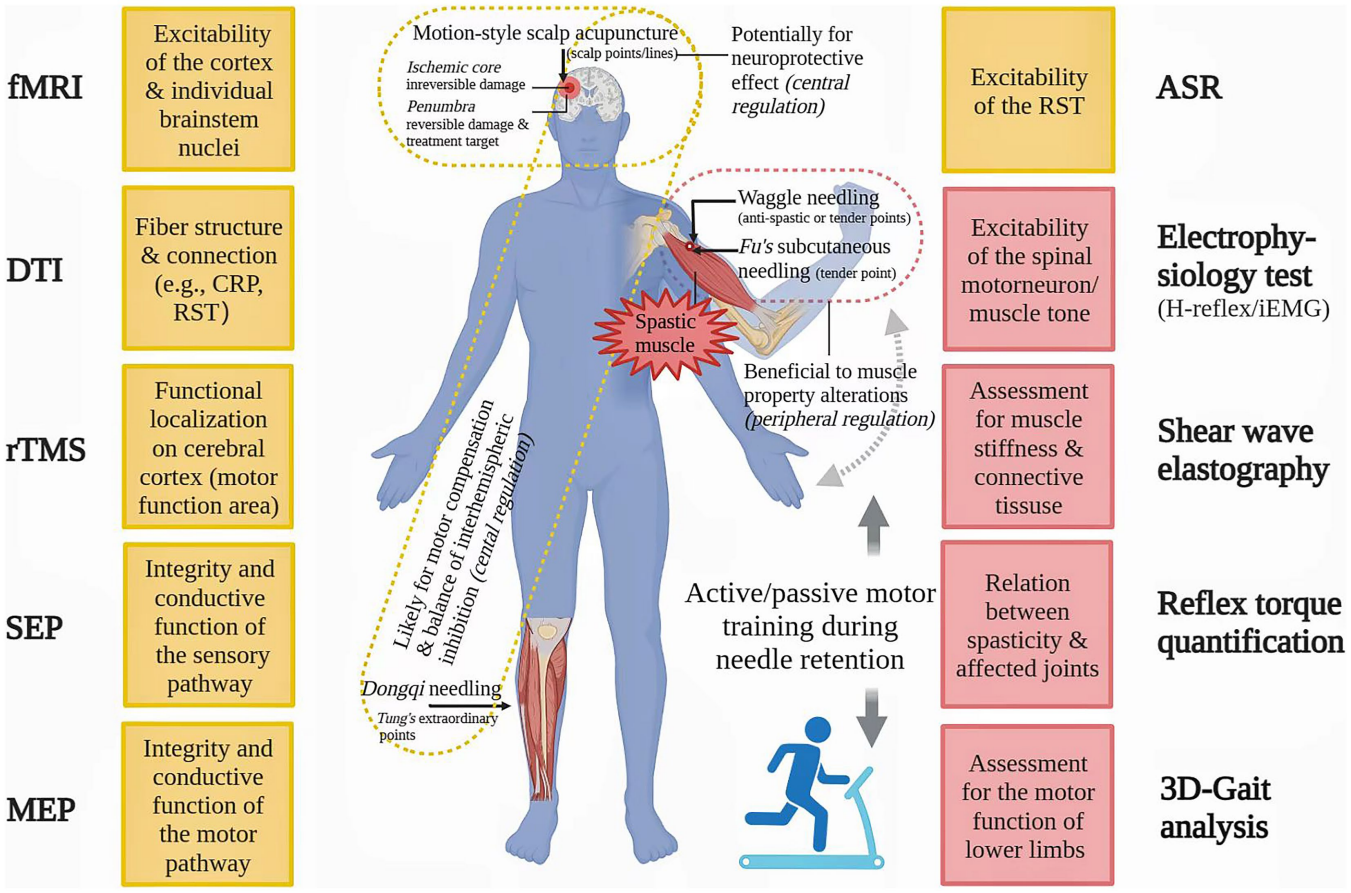
Advanced methods for spasticity assessment and their potential application in future motion-style acupuncture research. ASP: acoustic startle reflex, CRP: corticoreticular pathway, DTI: diffusion tensor imaging, fMRI: functional magnetic resonance imaging, iEMG: integrated electromyography, MEP: motor evoked potential, rTMS: repetitive transcranial magnetic stimulation, RST: reticulospinal tract, SEP: somatosensory evoked potential.

For better verification of the efficacy of MSA techniques on PSS, we propose that controls such as routine needling and sham needling, should be designed in future clinical studies, including patient’s self-report of needle sensation as well as objective measurements which may optimize clinical outcomes and improve patient adherence (130–132). Use of valid and reliable assessment tools, such as iEMG and shear wave elastography (133), could produce safe and timely evaluation on peripheral muscular changes. Functional magnetic resonance imaging (fMRI) (6, 86) and repetitive transcranial magnetic stimulation (rTMS) (134), could evaluate the excitability of individual brainstem nuclei and cortex in real-time respectively, and thereby provide direct evidences for neuromodulation mechanisms of MSA against PSS. Diffusion tensor imaging (DTI) can help us to observe the microstructural changes of fibers like CRP (40). In addition, the acoustic startle reflex (ASR), a brainstem-mediated reflex *via* RST, is also a good way to examine RST excitability non-invasively (135). Certain objective methods for PSS assessment and their prospective applications in MSA studies are exhibited in Figure 2.

## Conclusion

With the trend of aging population worldwide getting more obvious, the incidence of stroke is increasingly high. Spasticity, as a major medical problem following stroke, usually leads to abnormal posture and movement patterns, and greatly reduces the quality of life in patients. Unfortunately, the pathogenesis of PSS has not been fully understood till now, and its ideal and specific treatment regimen is deficient. Previous studies have demonstrated that occurrence of spasticity correlates with abnormalities in the central modulation and muscular adjustment. Facing the complicated situation, multidisciplinary approaches that could reconcile the central with the peripheral is considered as a noteworthy insight for doctors and researchers in the management of spasticity. As a portion of comprehensive remedy of PSS, MSA techniques, which are unique in that effective and synchronous combination of acupuncture and rehabilitation, have shown promising practical values in the clinical settings, with meaningful reduction in spastic hypertonia and improvement in motor performance, which may correlate with improvements of neuroplasticity and muscle condition. In order to ensure the effectiveness of treatment, it is necessary to consider not only the intervention timing and course, choice of needling technique, stimulation intensity, acupoint-selection (local/remote, bilateral/unilateral), exercise mode, predictive/aggravating factors of PSS, but the specific conditions (e.g., poststroke phases, extent of neurological deficit) and tolerance of patients (e.g., fatigue tolerance, pain tolerance). To avoid decreased efficacy and tolerance of acupuncture induced by frequent application of traditional acupoints and the same needling technique, using MSA techniques alternatively can be regarded as a feasible choice, especially suitable for those with long and complex disease progression. We hope this review can provide certain new insights for future research on acupuncture against PSS. Given that the quality of the current MSA studies is not optimal, rigorous study design, valid assessment tools for spasticity and related animal studies are expected to provide more substantial scientific evidences for MSA studies.

## Author contributions

J-XW wrote and revised the manuscript, also drew the figures and table. L-XM revised the manuscript. J-XW and OF checked the language together. All authors contributed to the article and approved the submitted version.

## Funding

This study was supported by the Fundamental Research Funds for the Central Universities of China (2022-JYB-JBZR-037 and 2022-JYB-XJSJJ-038).

## Conflict of interest

The authors declare that the research was conducted in the absence of any commercial or financial relationships that could be construed as a potential conflict of interest.

## Publisher’s note

All claims expressed in this article are solely those of the authors and do not necessarily represent those of their affiliated organizations, or those of the publisher, the editors and the reviewers. Any product that may be evaluated in this article, or claim that may be made by its manufacturer, is not guaranteed or endorsed by the publisher.

## References

[1] Feigin VL Vos T Nichols E Owolabi MO Carroll WM Dichgans M. The global burden of neurological disorders: translating evidence into policy. Lancet Neurol. (2020) 19:255–65. doi: 10.1016/S1474-4422(19)30411-9, PMID: 31813850 PMC9945815

[2] Li S Francisco GE Rymer WZ. A new definition of Poststroke spasticity and the interference of spasticity with motor recovery from acute to chronic stages. Neurorehabil Neural Repair. (2021) 35:601–10. doi: 10.1177/15459683211011214, PMID: 33978513

[3] Zeng H Chen J Guo Y Tan S. Prevalence and risk factors for spasticity after stroke: a systematic review and meta-analysis. Front Neurol. (2021) 11:616097. doi: 10.3389/fneur.2020.616097, PMID: 33551975 PMC7855612

[4] Wissel J Manack A Brainin M. Toward an epidemiology of poststroke spasticity. Neurology. (2013) 80:S13–9. doi: 10.1212/WNL.0b013e3182762448, PMID: 23319481

[5] Li S. Spasticity, motor recovery, and neural plasticity after stroke. Front Neurol. (2017) 8:120. doi: 10.3389/fneur.2017.00120, PMID: 28421032 PMC5377239

[6] Li S Francisco GE. New insights into the pathophysiology of post-stroke spasticity. Front Hum Neurosci. (2015) 9:192. doi: 10.3389/fnhum.2015.00192, PMID: 25914638 PMC4392691

[7] Lundström E Smits A Borg J Terént A. Four-fold increase in direct costs of stroke survivors with spasticity compared with stroke survivors without spasticity: the first year after the event. Stroke. (2010) 41:319–24. doi: 10.1161/STROKEAHA.109.558619, PMID: 20044535

[8] Francisco GE McGuire JR. Poststroke spasticity management. Stroke. (2012) 43:3132–6. doi: 10.1161/STROKEAHA.111.639831 22984012

[9] Winstein CJ Stein J Arena R Bates B Cherney LR Cramer SC. Guidelines for adult stroke rehabilitation and recovery: a guideline for healthcare professionals from the American Heart Association/American Stroke Association. Stroke. (2016) 47:e98–e169. doi: 10.1161/STR.0000000000000098, PMID: 27145936

[10] Thibaut A Chatelle C Ziegler E Bruno MA Laureys S Gosseries O. Spasticity after stroke: physiology, assessment and treatment. Brain Inj. (2013) 27:1093–105. doi: 10.3109/02699052.2013.804202 23885710

[11] Katalinic OM Harvey LA Herbert RD. Effectiveness of stretch for the treatment and prevention of contractures in people with neurological conditions: a systematic review. Phys Ther. (2011) 91:11–24. doi: 10.2522/ptj.20100265, PMID: 21127166

[12] MacPherson H Thomas K Walters S Fitter M. A prospective survey of adverse events and treatment reactions following 34,000 consultations with professional acupuncturists. Acupunct Med. (2001) 19:93–102. doi: 10.1136/aim.19.2.93, PMID: 11829165

[13] Wang HQ Hou M Bao CL Min L Li H. Effects of acupuncture treatment on lower limb spasticity in patients following hemorrhagic stroke: a pilot study. Eur Neurol. (2019) 81:5–12. doi: 10.1159/000499133, PMID: 31013499

[14] Li H Liu H Liu C Shi G Zhou W Zhao C. Effect of "Deqi" during the study of needling "Wang's Jiaji" acupoints treating spasticity after stroke. Evid Based Complement Alternat Med. (2014) 2014:715351. doi: 10.1155/2014/715351, PMID: 25477996 PMC4247953

[15] Wang BH Lin CL Li TM Lin SD Lin JG Chou LW. Selection of acupoints for managing upper-extremity spasticity in chronic stroke patients. Clin Interv Aging. (2014) 9:147–56. doi: 10.2147/CIA.S53814, PMID: 24453485 PMC3894143

[16] Zhu W Ye Y Liu Y Wang XR Shi GX Zhang S. Mechanisms of acupuncture therapy for cerebral ischemia: an evidence-based review of clinical and animal studies on cerebral ischemia. J Neuroimmune Pharmacol. (2017) 12:575–92. doi: 10.1007/s11481-017-9747-4, PMID: 28527041

[17] Qian X Ma LX Sun TY Mu JD Zhang Z Yu WY. Practical value and thought on "co-regulation of body and mind" in treatment of post-stroke spasticity with acupuncture. Zhongguo Zhen Jiu. (2022) 42:803–6. doi: 10.13703/j.0255-2930.20210718-k0003, PMID: 35793892

[18] Xue C Jiang C Zhu Y Liu X Zhong D Li Y. Effectiveness and safety of acupuncture for post-stroke spasticity: a systematic review and meta-analysis. Front Neurol. (2022) 13:942597. doi: 10.3389/fneur.2022.942597, PMID: 36062002 PMC9428153

[19] Neal E. Introduction to *Neijing* classical acupuncture part III: clinical therapeutics. J Chin Med. (2014) 104:5–23.

[20] Wang JX Ma LX Mohammad Reza AF Mohammadi A. Use of specific acupuncture techniques in lingering nummular eczema: a case report. J Tradit Chin Med Sci. (2021) 8:166–70. doi: 10.1016/j.jtcms.2021.03.001

[21] Qiu X Gao Y Zhang Z Cheng S Zhang S. Fire acupuncture versus conventional acupuncture to treat spasticity after stroke: a systematic review and meta-analysis. PLoS One. (2021) 16:e0249313. doi: 10.1371/journal.pone.0249313, PMID: 33836008 PMC8034732

[22] Zou DH Liu T Wang HB Chang H Li JN Li Q. Discussion on clinical application of "touching-bone" acupuncture technique. Zhongguo Zhen Jiu. (2020) 40:54–7. doi: 10.13703/j.0255-2930.20181210-0005, PMID: 31930900

[23] SCHILLEBEECKX F de GROEF A de BEUKELAER N DESLOOVERE K VERHEYDEN G PEERS K. Muscle and tendon properties of the spastic lower leg after stroke defined by ultrasonography: a systematic review. Eur J Phys Rehabil Med. (2021) 57:495–510. doi: 10.23736/S1973-9087.20.06462-X, PMID: 33305547

[24] Shi GX Liu BZ Wang J Fu QN Sun SF Liang RL. Motion style acupuncture therapy for shoulder pain: a randomized controlled trial. J Pain Res. (2018) Volume 11:2039–50. doi: 10.2147/JPR.S161951, PMID: 30310308 PMC6165767

[25] Kim D Park KS Lee JH Ryu WH Moon H Park J. Intensive motion style acupuncture treatment (MSAT) is effective for patients with acute whiplash injury: a randomized controlled trial. J Clin Med. (2020) 9:2079. doi: 10.3390/jcm9072079, PMID: 32630663 PMC7408694

[26] Shin JS Ha IH Lee J Choi Y Kim MR Park BY. Effects of motion style acupuncture treatment in acute low back pain patients with severe disability: a multicenter, randomized, controlled, comparative effectiveness trial. Pain. (2013) 154:1030–7. doi: 10.1016/j.pain.2013.03.013, PMID: 23639822

[27] Zhang Y Ma TM Bai ZH Sun BW Zhao HY. Meta-analysis on the therapeutic effect of acupuncture at Meridian sinew for spastic paralysis after stroke. Zhen Ci Yan Jiu. (2017) 42:178–82. 29071970

[28] Yan RJ Cheng B Chen LS Shen XY Zong L. Waggle needling plus joint needling for post-stroke spastic hemiplegia: a randomized controlled trail. Shanghai J Acupunct Moxib. (2016) 35:930–4. doi: 10.13460/j.issn.1005-0957.2016.08.0930

[29] Li XH Gan JX. Therapeutic effects of waggle needling at meridian-muscle nodes for spastic hemiplegia after stroke: a randomized controlled trail. Chin J Integr Med Cardio-Cerebrovasc Dis. (2017) 15:858–60.

[30] Chen JA Yu KC Zhong Z Zheng Y Qu SS Huang Y. Effect of Tung's acupuncture plus rehabilitation on stroke patients with upper limb spastic hemiplegia. Chin J Rehabil Theory Pract. (2015) 31:330–3.

[31] Chen JA Zhen Y Yu KC Zhong Z Qu SS Huang Y. Impacts of Dongqi needling at Tung’s extraordinary points integrated with motor training on the spastic upper limb paralysis in stroke patients: a randomized controlled trail. Chin J Rehabil Med. (2015) 30:715–7.

[32] Wang XY Wen X Zeng KX Liu T. Fu’s subcutaneous needling at starting and ending points of spastic muscle for elbow flexion spasticity in stroke survivors: a randomized controlled trail. Chin J Ethnomed Ethnopharm. (2018) 27:77–9.

[33] Zhang SH Wang YL Zhang CX Zhang CP Xiao P Li QF. Effects of interactive dynamic scalp acupuncture on motor function and gait of lower limbs after stroke: a multicenter, randomized, controlled clinical trial. Chin J Integr Med. (2022) 28:483–91. doi: 10.1007/s11655-021-3525-0, PMID: 34913147

[34] Zhang CX Wang YL Zhang SH Li QF Liang WR Pan XH. Impacts of motion-style scalp acupuncture on poststroke upper-limb spasticity: a randomized controlled trail. Shanghai J Acupunct-Mox. (2021) 40:937–44. doi: 10.13460/j.issn.1005-0957.2021.08.0937

[35] Li ZX Zhang XX Lou HJ Cong DY. Interactive scalp acupuncture for spastic paralysis after stroke: systemic review and meta-analysis. Lishizhen Med Materia Medica Res. (2021) 32:1510–4.

[36] Sangari S Perez MA. Imbalanced Corticospinal and Reticulospinal contributions to spasticity in humans with spinal cord injury. J Neurosci. (2019) 39:7872–81. doi: 10.1523/JNEUROSCI.1106-19.2019, PMID: 31413076 PMC6774405

[37] Mukherjee A Chakravarty A. Spasticity mechanisms - for the clinician. Front Neurol. (2010) 1:149. doi: 10.3389/fneur.2010.00149, PMID: 21206767 PMC3009478

[38] Burke D Wissel J Donnan GA. Pathophysiology of spasticity in stroke. Neurology. (2013) 80:S20–6. doi: 10.1212/WNL.0b013e31827624a7 23319482

[39] Gracies JM. Pathophysiology of spastic paresis. II: emergence of muscle overactivity. Muscle Nerve. (2005) 31:552–71. doi: 10.1002/mus.20285, PMID: 15714511

[40] Ko SH Kim T Min JH Kim M Ko HY Shin YI. Corticoreticular pathway in post-stroke spasticity: a diffusion tensor imaging study. J Pers Med. (2021) 11:1151. doi: 10.3390/jpm11111151, PMID: 34834503 PMC8621009

[41] Trompetto C Marinelli L Mori L Pelosin E Currà A Molfetta L. Pathophysiology of spasticity: implications for neurorehabilitation. Biomed Res Int. (2014) 2014:354906. doi: 10.1155/2014/354906, PMID: 25530960 PMC4229996

[42] Schreiner LH Lindsley DB Magoun HW. Role of brain stem facilitatory systems in maintenance of spasticity. J Neurophysiol. (1949) 12:207–16. doi: 10.1152/jn.1949.12.3.207, PMID: 18144617

[43] Li S Chen YT Francisco GE Zhou P Rymer WZ. A unifying pathophysiological account for post-stroke spasticity and disordered motor control. Front Neurol. (2019) 10:468. doi: 10.3389/fneur.2019.00468, PMID: 31133971 PMC6524557

[44] Katz RT Rymer WZ. Spastic hypertonia: mechanisms and measurement. Arch Phys Med Rehabil. (1989) 70:144–55. 2644919

[45] Nielsen JB Crone C Hultborn H. The spinal pathophysiology of spasticity--from a basic science point of view. Acta Physiol (Oxf). (2007) 189:171–80. doi: 10.1111/j.1748-1716.2006.01652.x, PMID: 17250567

[46] Sist B Fouad K Winship IR. Plasticity beyond peri-infarct cortex: spinal up regulation of structural plasticity, neurotrophins, and inflammatory cytokines during recovery from cortical stroke. Exp Neurol. (2014) 252:47–56. doi: 10.1016/j.expneurol.2013.11.019, PMID: 24291254

[47] Wang JX Yang X Zhang JJ Zhou TT Zhu YL Wang LY. Effects of Shaoyao Gancao decoction on contents of amino acids and expressions of receptors in brains of spastic paralysis rats. Zhongguo Zhong Yao Za Zhi. (2016) 41:1100–6. doi: 10.4268/cjcmm20160621, PMID: 28875677

[48] Kowalczyk P Kulig K. GABA system as a target for new drugs. Curr Med Chem. (2014) 21:3294–309. doi: 10.2174/0929867321666140601202158 24934345

[49] Gilbert SL Zhang L Forster ML Anderson JR Iwase T Soliven B. Trak1 mutation disrupts GABA(a) receptor homeostasis in hypertonic mice. Nat Genet. (2006) 38:245–50. doi: 10.1038/ng1715, PMID: 16380713

[50] Wang JX Ma LX Yang Y Song Y. Research progress on mechanism of acupuncture for the treatment of post-stoke spasticity. Global Tradit Chin Med. (2019) 12:470–5.

[51] Flores AE Pascotini ET Kegler A Broetto N Gabbi P Duarte T. Worst spasticity in patients post-stroke associated with MNSOD ALA16VAL polymorphism and interleukin-1β. Gene. (2022) 847:146880. doi: 10.1016/j.gene.2022.146880, PMID: 36100117

[52] Qi YC Xiao XJ Duan RS Yue YH Zhang XL Li JT. Effect of acupuncture on inflammatory cytokines expression of spastic cerebral palsy rats. Asian Pac J Trop Med. (2014) 7:492–5. doi: 10.1016/S1995-7645(14)60081-X, PMID: 25066401

[53] Lieber RL Steinman S Barash IA Chambers H. Structural and functional changes in spastic skeletal muscle. Muscle Nerve. (2004) 29:615–27. doi: 10.1002/mus.20059 15116365

[54] Jin Y Jin X Li J. Fu's subcutaneous needling and constraint-induced movement therapy for a patient with chronic stroke: one-year follow-up case report. Medicine. (2019) 98:e13918. doi: 10.1097/MD.0000000000013918, PMID: 30813122 PMC6408031

[55] Fu ZH Chen XY Lu LJ Lin J Xu JG. Immediate effect of Fu's subcutaneous needling for low back pain. Chin Med J. (2006) 119:953–7. doi: 10.1097/00029330-200606010-00014, PMID: 16780777

[56] Wang XW Yu XZ. Waggle needling combines with wheat-moxibustion for flexor surface electromyography and upper limb F wave in stroke patients with spastic paralysis: a randomized controlled trail. Acta Chin Med. (2017) 32:2558–61. doi: 10.16368/j.issn.1674-8999.2017.12.664

[57] Fu ZH Wang JH Sun JH Chen XY Xu JG. Fu's subcutaneous needling: possible clinical evidence of the subcutaneous connective tissue in acupuncture. J Altern Complement Med. (2007) 13:47–52. doi: 10.1089/acm.2006.6125, PMID: 17309377

[58] Ge RJ Hu XS Zeng KX. Preliminary exploration on the effect and mechanism of Dongqi needling at extraordinary points for spastic lower limb paralysis in stroke patients. J Liaoning Univ Tradit Chin Med. (2022) 24:140–5. doi: 10.13194/j.issn.1673-842x.2022.07.031

[59] Min YJ Yao HH Shao SJ He XW Wang HS Yan ZG. Brief analysis on scientifity of the international scalp acupuncture. Zhongguo Zhen Jiu. (2007) 27:612–6. 17853763

[60] Tao R Yin HN Liu SL Lv XL Zeng XX Sun ZR. Waggle needling for knee hyperextension during spastic phases and neurotransmitters and inflammatory cytokines in stroke patients: a randomized controlled trail. Chin J Integr Med Cardio-Cerebrovscu Dis. (2021) 19:1569–72.

[61] Xiao AJ Xia YB Fu ZH Guo J Liang S. Review on the role of Fu's subcutaneous needling (FSN) in pain relieving. Zhongguo Zhen Jiu. (2013) 33:1143–6. 24617252

[62] Song WP Zhang SM Wang J Yang J. Fu’s subcutaneous needling combined with motor training for stroke patients with spastic hand: a randomized controlled trial. J Pract Tradit Chin Med. (2022) 38:1035–7.

[63] Dai MY Zhou MY Wu LX Yu KQ Xu R. Fu’s subcutaneous needling integrated with muscle activation therapy for triceps muscle spasm during stroke recovery period: a randomized controlled trail. New Chin Med. (2021) 53:124–7. doi: 10.13457/j.cnki.jncm.2021.11.033

[64] Liu SL Zhang HY Man HJ. Impacts of motion-style scalp acupuncture on neurological deficit, somatosensory evoked potential and motor ability in stroke patients with spastic paralysis: a randomized controlled trial. Mod J Integr Tradit Chin West Med. (2018) 27:929–33.

[65] Tang TJ Sun KX Deng RC. Motion-style scalp acupuncture for the temporal and spatial parameters of gait in patients with spastic cerebral palsy: a randomized controlled trail. Shanghai J Acupunct-Mox. (2016) 35:1190–3.

[66] Li JT Wu P Wang RH. Motion-style scalp acupuncture for stroke patients with spastic hemiplegia: a randomized controlled trail. Chin J Integr Med Cardio-Cerebrovesc Dis. (2020) 18:1297–300.

[67] Zhang J Zhu L Tang Q. Electroacupuncture with rehabilitation training for limb spasticity reduction in post-stroke patients: a systematic review and meta-analysis. Top Stroke Rehabil. (2021) 28:340–61. doi: 10.1080/10749357.2020.1812938, PMID: 32845210

[68] Qi LL Han ZX Zhou YX Jiang SY Shen M Lu JY. Dynamic scalp acupuncture combined with PNF therapy for upper limb motor impairment in ischemic stroke spastic hemiplegia. Zhongguo Zhen Jiu. (2018) 38:234–8. doi: 10.13703/j.0255-2930.2018.03.002, PMID: 29701038

[69] Shi GX Yang CY Wu MM Guan LP Wang LP Liu CZ. Muscle hypertonia after permanent focal cerebral ischemia in rats: a qualitative and quantitative behavioral and electrophysiological study. Int J Neurosci. (2013) 123:575–81. doi: 10.3109/00207454.2013.783578, PMID: 23509968

[70] Wang JX Mu JD Ma LX Sun TY Qian X Yu WY. Waggle needling wields preferable neuroprotective and anti-spastic effects on post-stroke spasticity rats by attenuating γ-aminobutyric acid transaminase and enhancing γ-aminobutyric acid. Neuroreport. (2020) 31:708–16. doi: 10.1097/WNR.0000000000001471, PMID: 32453018 PMC7289130

[71] Wang JX Ma LX Mu JD Sun TY Qian X Yu WY. Anti-spastic effect induced by waggle needling correlates with KCC2-GABAA pathway in post-stroke spasticity rats. Neurosci Lett. (2021) 750:135810. doi: 10.1016/j.neulet.2021.135810, PMID: 33705929

[72] Sun TY Ma LX Mu JD Zhang Z Yu WY Qian X. Acupuncture improves the structure of spastic muscle and decreases spasticity by enhancing GABA, KCC2, and GABAAγ2 in the brainstem in rats after ischemic stroke. Neuroreport. (2022) Publish Ahead of Print:399–407. doi: 10.1097/WNR.0000000000001798, PMID: 35594431

[73] Qian X Ma LX Mu JD Zhang Z Sun TY Yu WY. Study on the central mechanism of acupuncture for post-stroke spasticity based on the Na^+^/K^+^-ATPase-EAATs-Glu pathway. Zhen Ci Yan Jiu. (2022) 47:283–9. doi: 10.13702/j.1000-0607.20210922, PMID: 35486007

[74] Mu JD Ma LX Zhang Z Yu WY Sun TY Qian X. Acupuncture alleviates spinal hyperreflexia and motor dysfunction in post-ischemic stroke rats with spastic hypertonia via KCC2-mediated spinal GABA_A_ activation. Exp Neurol. (2022) 354:114027. doi: 10.1016/j.expneurol.2022.114027, PMID: 35245503

[75] Akai M Nishimura R Fujita N. The swimming test is effective for evaluating spasticity after contusive spinal cord injury. PLoS One. (2017) 12:e0171937. doi: 10.1371/journal.pone.0171937, PMID: 28182676 PMC5300247

[76] Ward NS Brown MM Thompson AJ Frackowiak RS. Neural correlates of outcome after stroke: a cross-sectional fMRI study. Brain. (2003) 126:1430–48. doi: 10.1093/brain/awg145, PMID: 12764063 PMC3717456

[77] Di Pino G Pellegrino G Assenza G Capone F Ferreri F Formica D. Modulation of brain plasticity in stroke: a novel model for neurorehabilitation. Nat Rev Neurol. (2014) 10:597–608. doi: 10.1038/nrneurol.2014.162, PMID: 25201238

[78] Floel A Hummel F Duque J Knecht S Cohen LG. Influence of somatosensory input on interhemispheric interactions in patients with chronic stroke. Neurorehabil Neural Repair. (2008) 22:477–85. doi: 10.1177/1545968308316388, PMID: 18645188 PMC4890542

[79] Coleman ER Moudgal R Lang K Hyacinth HI Awosika OO Kissela BM. Early rehabilitation after stroke: a narrative review. Curr Atheroscler Rep. (2017) 19:59. doi: 10.1007/s11883-017-0686-6, PMID: 29116473 PMC5802378

[80] Jackman K Iadecola C. Neurovascular regulation in the ischemic brain. Antioxid Redox Signal. (2015) 22:149–60. doi: 10.1089/ars.2013.5669, PMID: 24328757 PMC4281847

[81] Carmichael ST. Cellular and molecular mechanisms of neural repair after stroke: making waves. Ann Neurol. (2006) 59:735–42. doi: 10.1002/ana.20845, PMID: 16634041

[82] Wei L Erinjeri JP Rovainen CM Woolsey TA. Collateral growth and angiogenesis around cortical stroke. Stroke. (2001) 32:2179–84. doi: 10.1161/hs0901.094282, PMID: 11546914

[83] Hong M Kim M Kim TW Park SS Kim MK Park YH. Treadmill exercise improves motor function and short-term memory by enhancing synaptic plasticity and neurogenesis in Photothrombotic stroke mice. Int Neurourol J. (2020) 24:S28–38. doi: 10.5213/inj.2040158.079, PMID: 32482055 PMC7285698

[84] Chen YT Li S DiTommaso C Zhou P Li S. Possible contributions of ipsilateral pathways from the Contralesional motor cortex to the voluntary contraction of the spastic elbow flexors in stroke survivors: a TMS study. Am J Phys Med Rehabil. (2019) 98:558–65. doi: 10.1097/PHM.000000000000114, PMID: 30672773 PMC6586481

[85] Nudo RJ Wise BM SiFuentes F Milliken GW. Neural substrates for the effects of rehabilitative training on motor recovery after ischemic infarct. Science. (1996) 272:1791–4. doi: 10.1126/science.272.5269.1791, PMID: 8650578

[86] Liu H Jiang Y Wang N Yan H Chen L Gao J. Scalp acupuncture enhances local brain regions functional activities and functional connections between cerebral hemispheres in acute ischemic stroke patients. Anat Rec (Hoboken). (2021) 304:2538–51. doi: 10.1002/ar.24746, PMID: 34431612 PMC9290874

[87] Schaechter JD Connell BD Stason WB Kaptchuk TJ Krebs DE Macklin EA. Correlated change in upper limb function and motor cortex activation after verum and sham acupuncture in patients with chronic stroke. J Altern Complement Med. (2007) 13:527–32. doi: 10.1089/acm.2007.6316, PMID: 17604556

[88] Dhond RP Kettner N Napadow V. Neuroimaging acupuncture effects in the human brain. J Altern Complement Med. (2007) 13:603–16. doi: 10.1089/acm.2007.7040 17718643

[89] Chen J Wang J Huang Y Lai X Tang C Yang J. Modulatory effect of acupuncture at Waiguan (TE5) on the functional connectivity of the central nervous system of patients with ischemic stroke in the left basal ganglia. PLoS One. (2014) 9:e96777. doi: 10.1371/journal.pone.0096777, PMID: 24927275 PMC4057077

[90] Howard JJ Herzog W. Skeletal muscle in cerebral palsy: from belly to myofibril. Front Neurol. (2021) 12:620852. doi: 10.3389/fneur.2021.620852, PMID: 33679586 PMC7930059

[91] Singer B Dunne J Allison G. Reflex and non-reflex elements of hypertonia in triceps surae muscles following acquired brain injury: implications for rehabilitation. Disabil Rehabil. (2001) 23:749–57. doi: 10.1080/09638280110060466, PMID: 11762877

[92] Kalkman BM Bar-On L O'Brien TD Maganaris CN. Stretching interventions in children with cerebral palsy: why are they ineffective in improving muscle function and how can we better their outcome? Front Physiol. (2020) 11:131. doi: 10.3389/fphys.2020.00131, PMID: 32153428 PMC7047287

[93] Miczak K Padova J. Muscle overactivity in the upper motor neuron syndrome: assessment and problem solving for complex cases: the role of physical and occupational therapy. Phys Med Rehabil Clin N Am. (2018) 29:529–36. doi: 10.1016/j.pmr.2018.03.006, PMID: 30626513

[94] Tian DS Xiong J Pan Q Liu F Wang L Xu SB. De qi, a threshold of the stimulus intensity, elicits the specific response of acupoints and intrinsic change of human brain to acupuncture. Evid Based Complement Alternat Med. (2014) 2014:914878. doi: 10.1155/2014/914878, PMID: 25228908 PMC4151069

[95] Yuan HW Ma LX Qi DD Zhang P Li CH Zhu J. The historical development of *Deqi* concept from classics of traditional Chinese medicine to modern research: exploitation of the connotation of *Deqi* in Chinese medicine. Evid Based Complement Alternat Med. (2013) 2013:639302. doi: 10.1155/2013/639302, PMID: 24302968 PMC3835614

[96] Shi GX Yang XM Liu CZ Wang LP. Factors contributing to therapeutic effects evaluated in acupuncture clinical trials. Trials. (2012) 13:42. doi: 10.1186/1745-6215-13-42, PMID: 22520963 PMC3404896

[97] Nierhaus T Chang Y Liu B Shi X Yi M Witt CM. Somatosensory stimulation with XNKQ acupuncture modulates functional connectivity of motor areas. Front Neurosci. (2019) 13:147. doi: 10.3389/fnins.2019.00147, PMID: 30914909 PMC6421982

[98] Konofagou EE Langevin HM. Using ultrasound to understand acupuncture. IEEE Pulse. (2005) 24:41–6. doi: 10.1109/memb.2005.1411347 15825844

[99] Lu Z Briley A Zhou P Li S. Are there trigger points in the spastic muscles? Electromyographical evidence of dry needling effects on spastic finger flexors in chronic stroke. Front Neurol. (2020) 11:78. doi: 10.3389/fneur.2020.00078, PMID: 32153489 PMC7047231

[100] Theodosiadou A Henry M Duchateau J Baudry S. Revisiting the use of Hoffmann reflex in motor control research on humans. Eur J Appl Physiol. (2022). doi: 10.1007/s00421-022-05119-7, PMID: 36571622

[101] Liu QG Liu L Huang QM Nguyen TT Ma YT Zhao JM. Decreased spontaneous electrical activity and acetylcholine at Myofascial trigger spots after dry needling treatment: a pilot study. Evid Based Complement Alternat Med. (2017) 2017:3938191–7. doi: 10.1155/2017/3938191, PMID: 28592980 PMC5448056

[102] Krakauer JW Carmichael ST Corbett D Wittenberg GF. Getting neurorehabilitation right: what can be learned from animal models? Neurorehabil Neural Repair. (2012) 26:923–31. doi: 10.1177/1545968312440745, PMID: 22466792 PMC4554531

[103] Li F Pendy JT Jr Ding JN Peng C Li X Shen J. Exercise rehabilitation immediately following ischemic stroke exacerbates inflammatory injury. Neurol Res. (2017) 39:530–7. doi: 10.1080/01616412.2017.1315882, PMID: 28415917

[104] Zhang Q Zhang J Yan Y Zhang P Zhang W Xia R. Proinflammatory cytokines correlate with early exercise attenuating anxiety-like behavior after cerebral ischemia. Brain Behav. (2017) 7:e00854. doi: 10.1002/brb3.854, PMID: 29201553 PMC5698870

[105] Zhang L Hu X Luo J Li L Chen X Huang R. Physical exercise improves functional recovery through mitigation of autophagy, attenuation of apoptosis and enhancement of neurogenesis after MCAO in rats. BMC Neurosci. (2013) 14:46. doi: 10.1186/1471-2202-14-46, PMID: 23565939 PMC3637142

[106] AVERT Trial Collaboration group. Efficacy and safety of very early mobilisation within 24 h of stroke onset (AVERT): a randomised controlled trial. Lancet. (2015) 386:46–55. doi: 10.1016/S0140-6736(15)60690-0, PMID: 25892679

[107] Momosaki R Yasunaga H Kakuda W Matsui H Fushimi K Abo M. Very early versus delayed rehabilitation for acute ischemic stroke patients with intravenous recombinant tissue plasminogen activator: a Nationwide retrospective cohort study. Cerebrovasc Dis. (2016) 42:41–8. doi: 10.1159/000444720, PMID: 26986718

[108] Wang Q Peng Y Chen S Gou X Hu B Du J. Pretreatment with electroacupuncture induces rapid tolerance to focal cerebral ischemia through regulation of endocannabinoid system. Stroke. (2009) 40:2157–64. doi: 10.1161/STROKEAHA.108.541490, PMID: 19372445

[109] Zhang BY Wang GR Ning WH Liu J Yang S Shen Y. Electroacupuncture pretreatment elicits tolerance to cerebral ischemia/reperfusion through inhibition of the GluN2B/m-Calpain/p38 MAPK Proapoptotic pathway. Neural Plast. (2020) 2020:8840675–14. doi: 10.1155/2020/8840675, PMID: 33061951 PMC7542475

[110] Gao Z Liu Q Yang L Zhu X. Identification of high-risk factors for prehospital delay for patients with stroke using the risk matrix methods. Front Public Health. (2022) 10:858926. doi: 10.3389/fpubh.2022.858926, PMID: 36438229 PMC9691690

[111] White A Cummings M Filshie J. (2008). An introduction to western medical acupuncture. London: Churchill Livingstone Elsevier. p.7–76.

[112] Tian L Wang JH Sun RJ Zhang XH Yuan B Du XZ. Development of researches on scalp acupuncture for ischemic stroke. Zhen Ci Yan Jiu. (2016) 41:93. 27141629

[113] Wang WW Xie CL Lu L Zheng GQ. A systematic review and meta-analysis of Baihui (GV20)-based scalp acupuncture in experimental ischemic stroke. Sci Rep. (2014) 4:3981. doi: 10.1038/srep03981, PMID: 24496233 PMC5379241

[114] Kelso JA Holt KG Rubin P Kugler PN. Patterns of human interlimb coordination emerge from the properties of non-linear, limit cycle oscillatory processes: theory and data. J Mot Behav. (1981) 13:226–61. doi: 10.1080/00222895.1981.10735251, PMID: 15215072

[115] Li A Yan T Chen Y. Impacts of unilateral needling and bilateral needling on the somatosensory evoked potential on patients with acute ischemic stroke: a randomized controlled trail. Chin J Rehabil Med. (2007) 22:81–2.

[116] Liu L Chen SQ Wei J Xu XB Jing XH Wang LP. The effect of acupuncture at Wang’s twelve points in the hands and feet on the plasticity of primary motor cortex in patients with ischemic stroke. Global Tradit Chin Med. (2019) 12:385–9.

[117] Shen TL. Influence of scalp acupuncture at unilateral side and bilateral sides on TCD in acute cerebral infarction: a randomized controlled trail. Shanghai J Acupunct-Mox. (2002) 1:8–10. doi: 10.13460/j.issn.1005-0957.2002.01.007

[118] McCombe Waller S Forrester L Villagra F Whitall J. Intracortical inhibition and facilitation with unilateral dominant, unilateral nondominant and bilateral movement tasks in left- and right-handed adults. J Neurol Sci. (2008) 269:96–104. doi: 10.1016/j.jns.2007.12.033, PMID: 18336839 PMC2910578

[119] Cauraugh JH Lodha N Naik SK Summers JJ. Bilateral movement training and stroke motor recovery progress: a structured review and meta-analysis. Hum Mov Sci. (2010) 29:853–70. doi: 10.1016/j.humov.2009.09.004, PMID: 19926154 PMC2889142

[120] Khan F Amatya B Bensmail D Yelnik A. Non-pharmacological interventions for spasticity in adults: an overview of systematic reviews. Ann Phys Rehabil Med. (2019) 62:265–73. doi: 10.1016/j.rehab.2017.10.001, PMID: 29042299

[121] Han P Zhang W Kang L Ma Y Fu L Jia L. Clinical evidence of exercise benefits for stroke. Adv Exp Med Biol. (2017) 1000:131–51. doi: 10.1007/978-981-10-4304-8_9 29098620

[122] Brusola G Garcia E Albosta M Daly A Kafes K Furtado M. Effectiveness of physical therapy interventions on post-stroke spasticity: an umbrella review. NeuroRehabilitation. (2023):1–15. doi: 10.3233/NRE-220275, PMID: 36806522

[123] Coronado RA Simon CB Valencia C George SZ. Experimental pain responses support peripheral and central sensitization in patients with unilateral shoulder pain. Clin J Pain. (2014) 30:143–51. doi: 10.1097/AJP.0b013e318287a2a4, PMID: 23619203 PMC3732495

[124] Zorowitz RD Gillard PJ Brainin M. Poststroke spasticity: sequelae and burden on stroke survivors and caregivers. Neurology. (2013) 80:S45–52. doi: 10.1212/WNL.0b013e3182764c86, PMID: 23319485

[125] Glaess-Leistner S Ri SJ Audebert HJ Wissel J. Early clinical predictors of post stroke spasticity. Top Stroke Rehabil. (2021) 28:508–18. doi: 10.1080/10749357.2020.1843845, PMID: 33156735

[126] Paul S Candelario-Jalil E. Emerging neuroprotective strategies for the treatment of ischemic stroke: an overview of clinical and preclinical studies. Exp Neurol. (2021) 335:113518. doi: 10.1016/j.expneurol.2020.113518, PMID: 33144066 PMC7869696

[127] Chen H He Y Chen S Qi S Shen J. Therapeutic targets of oxidative/nitrosative stress and neuroinflammation in ischemic stroke: applications for natural product efficacy with omics and systemic biology. Pharmacol Res. (2020) 158:104877. doi: 10.1016/j.phrs.2020.104877, PMID: 32407958

[128] Hu C Qin X Ye R Jiang M Lu Y Lin C. The role of traditional Chinese medicine nursing for stroke: an umbrella review. Evid Based Complement Alternat Med. (2021) 2021:9918687–12. doi: 10.1155/2021/9918687, PMID: 34306161 PMC8266461

[129] Huppertz V Guida S Holdoway A Strilciuc S Baijens L Schols JMGA. Impaired nutritional condition after stroke from the Hyperacute to the chronic phase: a systematic review and meta-analysis. Front Neurol. (2022) 12:780080. doi: 10.3389/fneur.2021.780080, PMID: 35178021 PMC8846185

[130] Briggs JP Shurtleff D. Acupuncture and the complex connections between the mind and the body. JAMA. (2017) 317:2489–90. doi: 10.1001/jama.2017.7214, PMID: 28654992

[131] Zhao L Liang FR Li Y Zhang FW Zheng H Wu X. Improved quality monitoring of multi-center acupuncture clinical trials in China. Trials. (2009) 10:123. doi: 10.1186/1745-6215-10-123, PMID: 20035630 PMC2806366

[132] Yuan HW Ma LX Zhang P Lin C Qi DD Li J. An exploratory survey of deqi sensation from the views and experiences of chinese patients and acupuncturists. Evid Based Complement Alternat Med. (2013) 2013:430851. doi: 10.1155/2013/430851, PMID: 24348700 PMC3857737

[133] Davis LC Baumer TG Bey MJ Holsbeeck MV. Clinical utilization of shear wave elastography in the musculoskeletal system. Ultrasonography. (2019) 38:2–12. doi: 10.14366/usg.18039, PMID: 30343557 PMC6323314

[134] Boddington LJ Reynolds JNJ. Targeting interhemispheric inhibition with neuromodulation to enhance stroke rehabilitation. Brain Stimul. (2017) 10:214–22. doi: 10.1016/j.brs.2017.01.006, PMID: 28117178

[135] Fawcett TJ Longenecker RJ Brunelle DL Berger JI Wallace MN Galazyuk AV. Universal automated classification of the acoustic startle reflex using machine learning. Hear Res. (2023) 428:108667. doi: 10.1016/j.heares.2022.108667, PMID: 36566642 PMC10734095

